# Comparison of facilitated tucking and oral dextrose in reducing the pain of heel stick in preterm infants: a randomized clinical trial

**DOI:** 10.1186/s12887-020-2020-7

**Published:** 2020-04-14

**Authors:** Athareh Ranjbar, Colleen Bernstein, Mamak Shariat, Hadi Ranjbar

**Affiliations:** 1 Tehran University of Medical Sciences, Tehran, Iran; 2 Department of Psychology, School of Human and Community Development, University of the Witwatersrand, Johannesburg, South Africa; 3 Materno-Fetal, Neonatal Research Center, Tehran University of Medical Sciences, Tehran, Iran; 4 Mental Health Research Center, Psychosocial Health Research Institute, Iran University of Medical Science, Tehran, Iran

**Keywords:** Pain, Blood sampling, Premature infant, Emergency department, Nurses

## Abstract

**Background:**

With the increase in hospitalization of premature infants in emergency departments and the painful procedure in these sectors, appropriate methods of pain relief are required. This study aimed to compare the effect of oral dextrose and facilitated tucking in the reduction of pain during heel sticks in premature infants and assess their effectiveness and feasibility for use in emergency settings.

**Methods:**

This study was a randomized controlled clinical trial with cross-over design. Sixty infants were recruited from a Neonatal Intensive Care Unit (NICU) at Valiasr hospital in Tehran, Iran from March 2015 to September 2016. They were randomly allocated into three groups (no pain relief method, oral dextrose and facilitated tucking). Six blood samples were collected by heel stick for each infant. Oral dextrose and facilitated tucking were compared with the routine method of blood sampling and pain was measured two times for each method. The pain scores was measured by the Premature Infant Pain Profile (PIPP). Repeated Measure ANOVA, ANOVA and Scheffe post-hoc test were used with SPSS 16.

**Results:**

The pain score’s increase during heel stick was significantly lower after using oral dextrose (3.58 ± 0.34) and facilitated tucking (5.58 ± 0.53) in comparison to the routine method (8.91 ± 0.18) of blood sampling (*P* < 0.001, η^2^ = 0.971). Oral dextrose was more effective than facilitated tucking (*P* < 0.001, Cohen’s d = 4.49). The emergency nurses rated oral dextrose as easier (t = 2.20, df = 118, *p* = 0.02, Cohen’s d = 0.39) and more applicable method (t = 2.99, df = 118, *p* = 0.003, Cohen’s d = 0.54) for the emergency department.

**Conclusions:**

Facilitated tucking is an effective method of pain reduction which can be used in the absence of oral dextrose, in a situation in which it is contraindicated or in combination with oral dextrose. Based on the increase of infant’s admission in emergency department future studies are needed to identify the best method of pain reduction for procedures in this setting.

**Trial registration:**

Current Controlled Trials IRCT201408029568N9, 2014-09-08.

## Background

Pain in newborns can cause severe problems in growth and development [1]. Procedural pain is one of the most frequent pains that infants may experience [2]. Recent research has shown that painful experiences can negatively impact infants’ brains with implications on their neurodevelopment and pain reactivity [3, 4]. However, pain relief methods are less likely to be used in the emergency departments [5]. Nurses in these settings usually do not have special skills or enough time to apply sophisticated methods of pain reduction.

With the increase in the survival rate of premature infants due to the advancement in technology, their admission rates in emergency departments have also increased. Between 2002 and 2012, there has been an increasing rate of admission rates at the rate of 3% of infants in emergency departments within the United States [6]. The result of a study showed that the rate of readmission of preterm neonates was 15.2%, which was significantly higher than term neonates (7.9%) who were hospitalized after birth [7]. The result of a review showed that the rate of emergency department visit of preterm neonates was higher than term ones [8]. It can be concluded that many emergency department admissions of neonates belong to premature neonates.

While we did not find related statistics of emergency admissions of premature infants in Iran. However, because of the fast development of critical care and increase of in premature births [7] we can assume conclude that in Iran also the rate of emergency department admissions of infants is has also increased in past years. With the increasing rate of admission of infants in emergency departments, the use of painful procedures has also increased.”. As such, we therefore need to find a practical method of pain control, that can be easily applied within emergency departments.

Although the present study focused on pre-term infants and not term infants, the development of pain sensation starts from the early life stages and affects the development of brain. The sensory receptors are present in neonates at 7 weeks of gestation. The cortical connections to process pain develop around 20–24 weeks of gestation. Therefore, descending pathways that inhibit pain are still functionally immature in term neonates which pain experienced could thus also cause changes in their brain development [9, 10]. It is therefore likely that both pre-term and term infants would react in the same manner to the pain of the heel stick itself and to the pain relief methods applied when doing heel sticks.

As more admissions of infants occur in emergency departments, painful procedures are also being carried out more and more in these areas [11]. Many palliative methods have been studied for children [12]. Also, pain relief in infants has been the subject of many studies [11, 13, 14]. However, methods that are merely applicable to the emergency department are less well considered. Most pain reduction methods require analgesic injections or applications which are not suitable for infants. Also, most non-pharmacological methods are time-consuming and are therefore not applicable within the emergency sector. Oral dextrose and facilitated tucking are two non-pharmacological methods that can be used in emergency departments. There are other methods like using a pacifier and breast milk which are safe and not sophisticated. However, they may not always be available in emergency settings. Oral dextrose and facilitated tucking are both inexpensive, quick and easy to use and accessible in every setting. They are also used widely in neonatal intensive care units. In the current study, we used these two methods, carried out by fully trained emergency nurses in neonatal intensive care units to assess their effectiveness and feasibility for use in emergency settings.

## Methods

### Design

The study adopted a randomized clinical trial with crossover design, to compare the effect of facilitated tucking and oral dextrose in the reduction of pain of routine heel sticks. Sixty infants needing heel stick procedures were randomly assigned to three groups. The primary outcome was the pain experienced during the heel stick which was measured using The Premature Infant Pain Profile (PIPP). The secondary outcome was the feasibility of the two methods which was assessed by two questions posed to emergency nurses. This study followed the CONSORT guidelines for reporting randomized controlled trials.

### Setting and sample

**Fig. 1:**
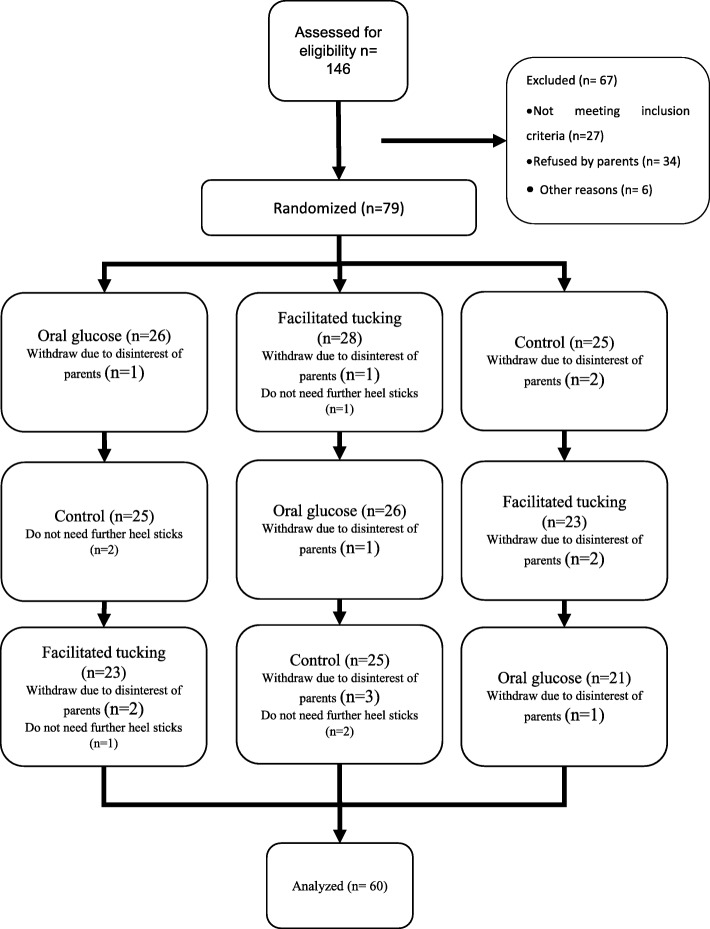
Study flowchart: recruitment and allocation to study groups

The convenience sampling method was used, and infants were randomly assigned to one of the three groups, with a blocking design. In this regard, the corresponding authors and the statistician were not present in the patient enrollment, and the first author was not involved in data analysis. We used a cross-over design and all samples received all treatments. The sample was recruited from a Neonatal Intensive Care Unit (NICU) at Valiasr hospital in Tehran, Iran from March 2015 to September 2016. Blood sampling in the NICU was based on physician order and based on patient circumstances. There was no routine method of pain reduction in the unit at the time of sampling. The sample recruitment continued to the minimum sample size achieved. Recruitment and allocation to study groups are presented in Fig. 1.

The minimum sample size was determined using the equation for comparing two means and parameters as follows: an alpha of 0.05, a power of 0.80, and a standard deviation from [15] 4.95 to discover a minimum difference of 2.93. Preterm infants with a gestational age between 28 to 36 weeks in NICU were considered eligible.

Inclusion criteria were (1) absence of asphyxia at birth based on infant records, (2) birth age of 2–28 days, (3) anticipated to have at least six heel sticks during the NICU hospitalization (4) no administration of sedatives relaxants, antiepileptics, or analgesic in 24 h before every study session, (5) born to mothers with no history of addiction to substances and (6) not having any acute condition which required more critical care surgery and the usage of sedative, relaxants, antiepileptics, or analgesic drugs.

### Data collection and processing

Infants who were expected to have six heel stick procedures were enrolled. We had three procedures which were used in a cross over design, therefore we should have had at least three measurements for each infant. To have a more accurate pain score we used two measurements per procedure and calculated the mean score of two measurements. The first author explained the purpose of the research and procedures to parents. Parents were informed that sampling is carried out according to the treatment process and that pain relief methods would only be applied with parental consent. Parents were also provided with a full description of the various pain relief methods and how levels of pain within each method would be measured. After full disclosure to parents about sampling, pain relief methods and measurement, written informed consent was obtained.

Heel sampling and the study intervention were always conducted by trained nurses who were the personnel of the emergency department. The first author assisted by another trained nurse completed the pain scale, and an interrater correlation of 0.92 was recorded. The first author and the trained nurse rated neonates separately on different sheets and they did not communicate during or after ratings. Infants with inclusion criteria were randomly allocated to three groups (A, B and C) by permutated blocks of six. Six blocks were defined (ABC, ACB, BAC, BCA, CAB and CBA) and a number between 1 and 6 were assigned to each block. By rolling a dice the sequence of blocks was determined. The process of assigning to groups, performing procedures and measuring within the three groups is presented in Fig. 1. All study subjects received both treatment and their pain recorded three times including heel stick with no intervention (Control).

Thirty minutes before the heel stick procedure infants were placed in a quiet location. The measurement of pain in the control measurements was performed without any pain relief intervention except the application of gentle touching and verbal comfort. Three nurses from emergency department did the procedures. The instructed nurses placed the babies in facilitated tucking position by placing them on their side, with their back gently bent, and their legs were in a flexion angle of greater than 90 degrees. The infants’ shoulders were also constricted up to 90 degrees, and the hands of the nurse were placed over the head close to the mouth or on the infant’s face. In the oral dextrose intervention, infants received 0.5 ml of 50% dextrose by a syringe 2 min before the procedure [2]. The same nurses performed all the six heel sticks for each of the infants in all six measurements. For more precision, in each step, the pain was measured across two heel sticks. The interval between the two measurements was never less than 2 hours. Infants who did not complete the six measurements were excluded from the study. All procedures were carried out by three nurses who worked in the emergency department.

### Feasibility

After each procedure, nurses were asked about the simplicity and the applicability of the methods. The questions were 1) how simple was the procedure; with scoring being between (1) very hard to (5) very simple and 2) how much they believe the procedure could be used within the emergency department with scoring between (1) not applicable to (5) fully applicable.

### Pain measurement

The Premature Infant Pain Profile (PIPP) was used as the outcome variable. PIPP scores were recorded at three times: before, during and 5 minutes after sampling for each infant. The PIPP is a behavioral measure of pain for premature infants. It includes seven indicators: 1) gestational age, 2) the behavioral state, 3) change in heart rate, 4) change in oxygen saturation, 5) brow bulge; 6) eyes squeeze and 7) nasolabial furrow. The scoring is presented in Table 1. In each phase raters observed each infant for 15 s for the behavioral state, change in heart rate, change in oxygen saturation and 30 s for brow bulge, eyes squeeze and nasolabial furrow. Heart rate and saturation were measured and recorded by an EKG monitor and pulse oximetry. The total score is the summation of all seven indicators, with a minimum of 0 and maximum of 21; the higher the score, the greater the pain behavior.

**Table 1:** Premature infant pain profile

Indicators	0	1	2	3
GA in weeks	≥ 36 weeks	32 to 35 weeks and days	28 to 31 weeks and 6 days	< 26 weeks
Alertness	Active	Quiet	Active	Quiet
	Awake	Awake	Sleep	Sleeping
	Open Eyes	Open eyes	Closed eyes	Closed eyes
	Facial movements present	No facial movements	Facial movements present	No facial movements
Maximal HR	↑ 0 to 4 bpm	↑ 5 to 14 bpm	↑ 15 to 24 bpm	↑ ≥25 bpm
Minimal Saturation	↓ 0 to 2.4%	↓2.5 to 4.9%	↓ 5 to 7.4%	↓ ≥7.5%
Frowned forehead	Absent	Minimal	Moderate	Maximal
Eyes squeezed	Absent	Minimal	Moderate	Maximal
Nasolabial furrow	Absent	Minimal	Moderate	Maximal

Absent is defined as 0 to 9% of the observation time; minimal, 10 to 39% of time; moderate 40 to 69% of the time; and maximal as 70% or more of the observation time

The data was entered into SPSS Version 16. The pain reported as mean ± SD. The Shapiro-Wilk test was used to test for normality of PIPP scores (*p* > 0.05). The change in pain scores was tested by ANOVA and Scheffe post-hoc test between three groups and repeated measurement within each one of them. The independent samples t-test was used to compare the utility of methods from perspective of nurses. ANOVA was used to evaluate the carry-over effect. Repeated measurement ANOVA was used to evaluate the period effect. The level of significance was set at *p* < 0.05 in all tests.

### Ethical consideration

The study protocol was approved by the Ethics Committee of the Ethics Committees of Tehran University of Medical Sciences (TUMS.REC.1395.25966). The trial is registered in the IRCT201408029568N9 Before participation in the study, written informed consent was obtained from each child’s primary guardian.

## Results

### Study subjects

The mean gestational ages of infants were 32.35 weeks with an SD of 2.81 weeks. The mean of weights was 2173.45 ± 413.57 g. The study sample consisted of 23 (38.34%) girls and 37 (61.66%) boys. The mean and SD of the infants Apgar score was 8.85 ± 0.35. The period and carry over effects were tested and presented in Table 2. Based on the results of the table all measurements were independent and there were no time and carry-over effect.

**Table 2:** The test for period and carry over effect

Intervention	Before Heel Stick	During Heel Stick	After Heel Stick	Time Effect
Oral Dextrose	4.05 ± 1.15	10.05 ± 2.42	6.97 ± 1.76	F (1.03, 2.07) = 0.623, *p* = 0.543
Facilitated tucking	3.89 ± 1.13	10.06 ± 2.47	6.56 ± 1.70	
Control	4.03 ± 0.99	9.95 ± 2.55	6.78 ± 1.67	
Carry-over Effect	F(2, 177) = 0.381, *p* = 0.683	F(2, 177) = 0.381, *p* = 0.683	F(2, 177) = 0.381, *p* = 0.683	

### PIPP score

**Fig. 2:**
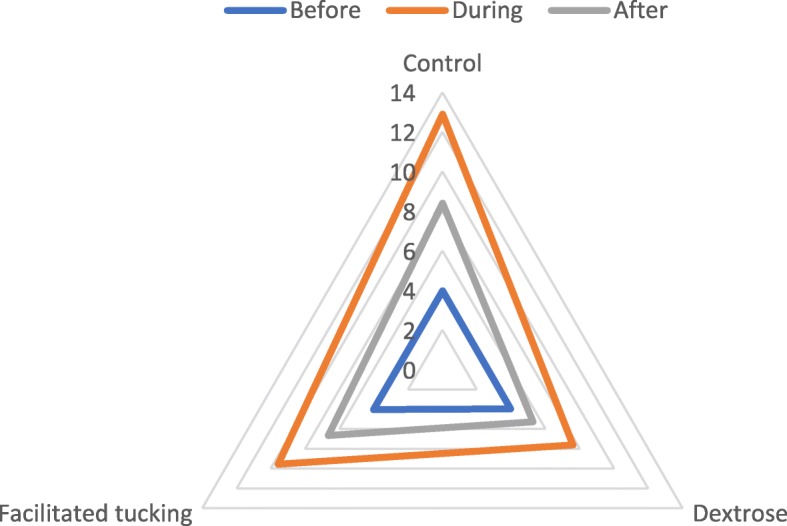
The PIPP score’s changes in three groups, before, during and after heel sticks

The results showed that the PIPP scores increased during the heel stick and decreased after that. The mean ± SD of PIPP scores before heel stick were 3.98 ± 1.13, 4.02 ± 1.10, 3.98 ± 1.06 for Oral Dextrose, facilitated tucking, control measurements, respectively (*p* = 0.97). The mean ± SD of PIPP scores during heel stick were 7.60 ± 1.17, 9.56 ± 1.15, 12.90 ± 1.14 for oral dextrose, facilitated tucking, control measurements, respectively (*p* < 0.001). The mean ± SD of PIPP scores after heel stick were 5.27 ± 1.19, 6.65 ± 1.09, 8.40 ± 1.14 for Oral Dextrose, facilitated tucking, control measurements, respectively (*p* < 0.001). The PIPP score’s changes are reported in Fig. 2. The PIPP scores changes are reported in Table 3. Post-hoc Scheffe tests showed that the increase of PIPP scores was lower after using oral dextrose in comparison to facilitated tucking and control groups. Also, the PIPP scores increased significantly in the control group in comparison to the facilitated tucking group. The decrease of PIPP scores after using the heel stick was higher in oral dextrose group in comparison to both the facilitated tucking and control groups. The decrease was also higher in the facilitated tucking group as compared to the control group. The multiple comparisons of PIPP score in different phases and groups are presented in Table 4.

**Table 3:** The comparison of PIPP scores in three methods, before, during and after heel stick

Measurement	Oral Dextrose (M ± SD)	Facilitated tucking (M ± SD)	Control (M ± SD)	ANOVA
Time				
Difference between during and before	3.58 ± 0.34	5.58 ± 0.53	8.91 ± 0.18	*p* < 0.001
Difference between after and during	−2.33 ± 0.23	−2.91 ± 0.53	−4.50 ± 0.29	*p* < 0.001
Difference between after and before	1.25 ± 0.38	2.66 ± 0.23	4.41 ± 0.18	*p* < 0.001

**Table 4:** The multiple comparisons of PIPP score in different phases and groups

PIPP Score	(I) Group	(J) Group	Mean Difference (I-J)	Sig.	95% Confidence Interval
Before heel stick	Control	Oral Dextrose	−0.04	0.98	[−0.53, 0.46]
		Facilitated tucking	0.00	1.00	(−0.50, 0.50)
	Oral Dextrose	Control	0.04	0.98	(−0.46, 0.53)
		Facilitated tucking	0.04	0.98	(−0.46, 0.53)
	Facilitated tucking	Control	0.00	1.00	(−0.50, 0.50)
		Oral Dextrose	−0.04	0.98	(−0.53, 0.46)
During heel stick	Control	Oral Dextrose	5.30	< 0.001	(4.77, 5.82)
		Facilitated tucking	3.33	< 0.001	(2.81, 3.85)
	Oral Dextrose	Control	−5.30	< 0.001	(−5.82, −4.77)
		Facilitated tucking	−1.96	< 0.001	(−2.48, −1.44)
	Facilitated tucking	Control	−3.33	< 0.001	(−3.85, − 2.81)
		Oral Dextrose	1.96	< 0.001	(1.44, 2.48)
After heel stick	Control	Oral Dextrose	3.13	< 0.001	(2.61, 3.64)
		Facilitated tucking	1.75	< 0.001	(1.24, 2.26)
	Oral Dextrose	Control	−3.13	< 0.001	(− 3.64, −2.61)
		Facilitated tucking	−1.38	< 0.001	(−1.89, −0.86)
	Facilitated tucking	Control	−1.75	< 0.001	(−2.26, − 1.24)
		Oral Dextrose	1.38	< 0.001	(0.86, 1.89)
During and before heel stick	Control	Oral Dextrose	5.33	< 0.001	(5.16, 5.51)
		Facilitated tucking	3.33	< 0.001	(3.16, 3.51)
	Oral Dextrose	Control	−5.33	< 0.001	(−5.51, −5.16)
		Facilitated tucking	−2.00	< 0.001	(−2.17, −1.83)
	Facilitated tucking	Control	−3.33	< 0.001	(−3.51, −3.16)
		Oral Dextrose	2.00	< 0.001	(1.83, 2.17)
After and during heel stick	Control	Oral Dextrose	−2.17	< 0.001	(− 2.34, − 2.00)
		Facilitated tucking	−1.58	< 0.001	(− 1.75, − 1.41)
	Oral Dextrose	Control	2.17	< 0.001	(2.00, 2.34)
		Facilitated tucking	0.58	< 0.001	(0.41, 0.75)
	Facilitated tucking	Control	1.58	< 0.001	(1.41, 1.75)
		Oral Dextrose	−0.58	< 0.001	(−0.75, −0.41)
After and before heel stick	Control	Oral Dextrose	3.17	< 0.001	(3.04, 3.29)
		Facilitated tucking	1.75	< 0.001	(1.62, 1.88)
	Oral Dextrose	Control	−3.17	< 0.001	(−3.29, −3.04)
		Facilitated tucking	−1.42	< 0.001	(−1.54, −1.29)
	Facilitated tucking	Control	−1.75	< 0.001	(−1.88, − 1.62)
		Oral Dextrose	1.42	< 0.001	(1.29, 1.54)

### Feasibility

The mean and standard deviation of the applicability score of the two methods were 4.26 ± 0.73 and 4.00 ± 0.58 respectively for oral dextrose and facilitated tucking (t = 2.20, df = 118, *p* = 0.02, Cohen’s d = 0.39). The mean and standard deviation of the easy-to-use score were 4.23 ± 0.72 and 3.83 ± 0.74, respectively, for oral dextrose and facilitated tucking (t = 2.99, df = 118, *p* = 0.003, Cohen’s d = 0.54).

## Discussion

The results of the current study showed that facilitated tucking and oral dextrose are both effective in reducing the pain of blood sampling in infants. These findings are aligned with the literature. For example, the results of another study showed that facilitated tucking was a suitable method for reducing pain in premature infants during blood sampling as compared to a control group [16]. However, within the present study oral dextrose led to significantly greater pain reduction as compared to facilitated tucking. It has been shown that oral dextrose has been effective in reducing the procedural pain in newborns [17–19].

Gradin and Schollin [20] argued orally administered dextrose has more significant effect because it reduces the pain of painful procedures by stimulating the secretion of endorphins, Similarly, Cohen, Blount, Chorney, Zempsky, Rodrigues and Cousins indicated that oral dextrose has a more significant impact on pain reduction [21]. This suggests that procedures such as oral administration of dextrose, due to stimulation of secretion of endorphins, are more effective in reduction of procedural pain.

Jatana, Dalal, and Wilson (2003) examined the effects of 10, 25 and 50% of oral dextrose, expressed breast milk and sterile water (the control group) on the amount of heel blood sampling pain in 125 infants. Their results indicate that the use of dextrose with different concentrations and expressed breast milk (EBM) has an effective analgesic result in full-term infants and can be used as a cost-effective method with many benefits and low side effects in reducing neonatal pain [22]. The results of the study by Golestan, Karbasi [23] showed that the pain response and duration of crying of newborns in the infants who received oral dextrose group (25 and 50%) before painful procedures was reduced more than infants who received 10% oral dextrose and EBM group [23].However, there was no significant difference between the two groups (25 and 50%). Further, all groups showed lower pain response and duration of crying as compared to the sterile water -control group.

Furthermore, the results of a study accomplished by Cignacco, Sellam [24], showed that the use of the facilitated tucking was not an effective way to relieve pain and they did not recommend it as a non-pharmacological intervention to relieve pain [24]. In the study by Liaw (2012), two methods of relieving the pain caused by blood sampling through heel stick of premature infants were compared. Those methods were non-nutritive sucking and facilitated tucking. The results of this study showed that both methods had better results in comparison with routine procedures. But non-nutritive sucking was more effective for relieving pain in comparison with facilitated tucking [15]. In another study by Liaw (2013), the effect of different combinations of non-nutritive sucking, oral dextrose and facilitated tucking on the sleep-wake state before, during and after heel stick was studied. They found that in order to keep babies’ asleep, caregivers should combine non-nutritional sucking, oral sucrose, and facilitated tucking to reduce restlessness during painful procedures [25].

A study was conducted to compare the effects of non-prescription pain relief methods on neonatal pain, physiological parameters and crying time before, during and after muscular injection of hepatitis B vaccine. The results of this study indicated that the use of both non-nutritional sucking and oral dextrose methods effectively relieves pain in infants, decrease the physiological parameters including heart rate and respiratory rate, and the duration of crying during the vaccine injection as compared to usual care [26]. The results of a systematic review in 2009, also, showed that facilitated tucking might be effective in preventing pain in painful procedures [27].

### Limitations

The main limitation of the current study was differences between the sampling setting and an actual emergency department. Neonatal intensive care units are very controlled setting. The use of cross-over design to control factors which affect pain needs several blood samplings, a situation which is likely not to occur in an emergency situation. Therefore, the only setting that was possible for this design in which multiple samplings could be assessed was within a NICU. However, we did use nurses from the emergency department to assess the feasibility of each method for usage within emergency departments. Based on their responses to the methods used we recommend using these methods in real life emergency situations to assess their simplicity and applicability.

Another limitation is the recruitment from neonates with age of gestation between 28 to 36 weeks. This can cause limitation in the generalizability of the results. However, because the pain pathways in term and preterm neonates may be very similar, clinicians can benefit from our results. While rating the scores was conducted independently by two rater, because of the simultaneous conduction of rating it may effect the scores, which should consider in future studies.

### Implications for emergency nurses

The results of previous studies have shown that these methods are effective primarily in NICU, but they did not consider them for use in the emergency department. We chose these methods because they are easy to use and they do not need complicated skills. These features make them feasible methods in emergency departments. Nurses and paramedics in the emergency sector prefer methods which are not time-consuming and with maximum effectiveness. Feasible methods for the emergency department should be easy to accomplish and not time-consuming. Facilitated tucking and oral dextrose are very easy to use methods, and they do not need special skills. Our results suggest that these methods may well be feasible in emergency settings as by including emergency nurses in our study and asking them to evaluate these methods in terms of their feasibility we did demonstrate that emergency nurses who trained to use these methods ranked them as easy and applicable to use in their work settings.

## Conclusion

The results of the current study indicate that facilitated tucking and oral dextrose are two effective methods of pain reduction which can use in Emergency settings. Oral dextrose was more effective in the reduction of pain and as it needs no specific training, we can therefore recommend it for use in emergency departments. The evaluation by emergency nurses within this study supports our recommendation. With the increase of the incidence of admission of infants to emergency departments and application of painful procedures with diagnostic and therapeutic indications, pain reduction methods should develop to address the need of pain relieve in these settings. Our results also showed that nurses with experience of work in ED rate that facilitated tucking and oral dextrose as too easy to use and applicable methods. These results also support these two methods of pain reduction to use in the emergency department.

## Data Availability

All data will be available on request. Everyone can request the data. To gain access, data requestors will need to sign a data access agreement. The data is available for any purpose. All applications should be sent to Ranjbar.h@iums.ac.ir. All requests will be answered within a maximum of 1 month by email.

## Abbreviations

ANOVA: Analysis of Variance
Bpm: Beat Per Minute
HR: Heart Rate
NICU: Neonatal Intensive Care Unit
PIPP: Premature Infant Pain Profile
